# Partial Reversibility of the Cytotoxic Effect Induced by Graphene-Based Materials in Skin Keratinocytes

**DOI:** 10.3390/nano10081602

**Published:** 2020-08-15

**Authors:** Marco Pelin, Hazel Lin, Arianna Gazzi, Silvio Sosa, Cristina Ponti, Amaya Ortega, Amaia Zurutuza, Ester Vázquez, Maurizio Prato, Aurelia Tubaro, Alberto Bianco

**Affiliations:** 1 Department of Life Sciences, University of Trieste, 34127 Trieste, Italy; ssosa@units.it (S.S.); cponti@units.it (C.P.); tubaro@units.it (A.T.); 2 CNRS, Immunology, Immunopathology and Therapeutic Chemistry, UPR 3572, University of Strasbourg, ISIS, 67000 Strasbourg, France; rhlin@ibmc-cnrs.unistra.fr; 3 Department of Chemical and Pharmaceutical Sciences, University of Trieste, 34127 Trieste, Italy; arianna.gazzi@phd.units.it (A.G.); prato@units.it (M.P.); 4 Fondazione Istituto di Ricerca Pediatrica, Città della Speranza, 35100 Padua, Italy; 5 Graphenea, 20009 Donostia-San Sebastián, Spain; a.ortega@graphenea.com (A.O.); a.zurutuza@graphenea.com (A.Z.); 6 Facultad de Ciencias y Tecnologías Químicas, Universidad de Castilla-La Mancha (UCLM), 13071 Ciudad Real, Spain; Ester.Vazquez@uclm.es; 7 Instituto Regional de Investigacion Científica Aplicada (IRICA), Universidad de Castilla-La Mancha, 13071 Ciudad Real, Spain; 8 Center for Cooperative Research in Biomaterials (CIC biomaGUNE), Basque Research and Technology Alliance (BRTA), Paseo de Miramon 182, 20014 Donostia San Sebastián, Spain; 9 Basque Foundation for Science (IKERBASQUE), 48013 Bilbao, Spain

**Keywords:** carbon nanomaterials, 2D materials, autophagy, biocompatibility, dermal toxicity

## Abstract

In the frame of graphene-based material (GBM) hazard characterization, particular attention should be given to the cutaneous effects. Hence, this study investigates if HaCaT skin keratinocytes exposed to high concentrations of few-layer graphene (FLG) or partially dehydrated graphene oxide (d-GO) for a short time can recover from the cytotoxic insult, measured by means of cell viability, mitochondrial damage and oxidative stress, after GBM removal from the cell medium. When compared to 24 or 72 h continuous exposure, recovery experiments suggest that the cytotoxicity induced by 24 h exposure to GBM is only partially recovered after 48 h culture in GBM-free medium. This partial recovery, higher for FLG as compared to GO, is not mediated by autophagy and could be the consequence of GBM internalization into cells. The ability of GBMs to be internalized inside keratinocytes together with the partial reversibility of the cellular damage is important in assessing the risk associated with skin exposure to GBM-containing devices.

## 1. Introduction

Graphene consists of a single atom thick, two-dimensional sheet of sp^2^–carbons arranged as six-membered rings in a honeycomb structure, identified in 2004 by scotch tape exfoliation of graphite [1,2]. Together with its derivatives (known as graphene-based materials; GBMs), such as few-layer graphene (FLG), graphene oxide (GO), reduced graphene oxide (rGO) and graphene nanoplatelets (GNPs), attention to graphene has significantly increased in recent years, due to its extraordinary physicochemical properties [3,4,5]. The intense research on GBMs is constantly evolving and has led to the development and study of numerous types of materials, but also to the development of various synthetic approaches for the production of high quality GBMs, guaranteeing their potential application in the most diverse areas of science and technology [6]. On the basis of their properties (i.e., high surface area, extraordinary electrical and thermal conductivity and strong mechanical strength), the current most promising applications of GBMs range from opto-electronics (electronic circuits, displays, supercapacitors, batteries and solar cells, to name a few) to biotechnology, such as tissue engineering, drug/gene delivery, innovative anticancer therapies, biosensing and bioimaging [7].

However, the use of GBMs in various technological fields requires the assessment of their potential impact on both human health and the environment. For this reason, the risk assessment of GBMs must be an integral part of the innovation process [8]. To date, the risk for human health associated with GBM exposure is mainly associated with an occupational scenario, related to both the industrial and small-scale production [9], but exposures associated with specific GBM applications should also be considered, given their entrance into the market. One of the most relevant exposure routes to GBMs is certainly the cutaneous one, although the associated risk is still rarely studied [8,9].

Compared to toxicity studies related to other exposure routes, the characterization of GBM toxicity after cutaneous exposure is based on rather limited data, derived from in vitro studies on skin fibroblasts [10], keratinocytes [11,12,13,14,15] and three-dimensional human epidermis [16]. Different exposure scenarios can be hypothesized for skin contact with GBM-based devices: (i) a continuous long-term exposure to GBMs, even at high concentrations, such as in the case of implantable devices; (ii) a long-term exposure, such as for workers exposed to GBMs; (iii) a short-term exposure, even alternated by periods without exposure, such as for exposure to wearable devices; (iv) accidental short-term exposure, such as for contact with GBM-enriched textiles or engineered apparatus (e.g., car seats). Using skin HaCaT keratinocytes, we previously demonstrated the impact of GBMs after both short (i.e., 24 h) and long (i.e., 72 h) exposures. In particular, GBMs with different physicochemical properties reduced cell viability by interaction with the plasma membrane, and that the cytotoxic potency is influenced by the material oxidation state and lateral dimension [12]. The cytotoxicity, involving a reactive oxygen species (ROS)-dependent mitochondrial dysfunction [13], was significant only after relatively long exposures (i.e., 72 h) to high GBM concentrations (>10 μg/mL) [12,13], suggesting a detrimental effect towards keratinocytes only after prolonged exposure to high GBM levels. However, in a subsequent study we demonstrated that keratinocytes are able to also sense low sub-cytotoxic concentrations of GBMs (<1 μg/mL) to initiate a pro-inflammatory response. Intriguingly, this effect was significant also after removal of the materials: keratinocytes exposed to low concentrations of FLG or GO (0.01–1.0 μg/mL) for a short exposure time (4 h) followed by longer culture in GBM-free medium (20 up to 68 h) still kept on releasing pro-inflammatory cytokines, and this release was significantly higher than that recorded after 24 or 72 h continued exposure to these materials [15]. This result suggests that, at least considering pro-inflammatory cytokine release, an initial GBM detection by skin keratinocytes is sufficient to activate these cells, and that GBM removal does not recover cells from the pro-inflammatory stimulus. However, similar data on the recovery of GBM-induced cytotoxicity, in particular at high GBM concentrations inducing far more important detrimental effects in keratinocytes, are not currently being reported. In the frame of GBM applications at the cutaneous level, in which devices (especially wearable ones) can be put directly into contact with the skin, these data could acquire an important role for the hazard characterization of GBMs at the cutaneous level. Therefore, the present study was carried out with the aim to investigate if HaCaT skin keratinocytes exposed to high GBM concentrations for a short time can recover from the cytotoxic insult through the physical removal of these materials. In addition, the role of autophagy, as one of the major cellular pathways to recover damaged cells, was evaluated.

## 2. Materials and Methods

### 2.1. Materials

FLG and dehydrated GO (herein named d-GO) were endotoxin-free to avoid false-positive results due to lipopolysaccharide (LPS) contamination. Both materials were the same as used in our previous study, in which full physicochemical characterization after endotoxin removal by heat treatment was carried out [15].

*Endotoxin-free FLG*. Endotoxin-free FLG was prepared by heat treatment of FLG powder, prepared through a solvent-free ball-milling under air atmosphere, as previously described [17,18]. Heat treatment consisted of a first heating ramp at 10 °C/min up to 129 °C, followed by a 60 min isothermal segment, and by a final cooling ramp (2 °C/min until 25 °C) using a TGA Q50 (TA Instruments, Madrid, Spain) under N_2_ conditions, as previously described [15].

*Endotoxin-free GO* (*d-GO*). Endotoxin-free GO was prepared by heat treatment of commercially available GO (Graphenea; San Sebastian, Spain) at 200 °C for 1 h in a protective Ar atmosphere. As previously demonstrated, since Raman and thermogravimetric analyses showed slight physicochemical alterations of GO samples after the heat treatment, ascribed to water loss, this material was accordingly renamed dehydrated GO (d-GO) [15].

### 2.2. Cell Culture and Exposure to Gbms

HaCaT cells are spontaneously immortalized non-tumor keratinocytes, cultured in high glucose Dulbecco’s Modified Eagle’s medium (DMEM), supplemented with 10% fetal bovine serum (FBS), 1.0 × 10^−2^ M L-glutamine, 1.0 × 10^−4^ g/mL penicillin and 1.0 × 10^−4^ g/mL streptomycin at 37 °C in a humidified 95% air/5% CO_2_ atmosphere. All reagents were purchased from Sigma-Aldrich (Milan. Italy). Cell passage was performed once a week and 2 days post-confluence. All the experiments were performed between passage 63 and 74. HaCaT cells were routinely checked for the absence of *Mycoplasma* infection.

Cells were exposed to GBMs (1–100 µg/mL) under different conditions: (i) continuous exposure for 24 or 72 h and (ii) recovery exposure, consisting of 24 h cell exposure to GBMs, followed by three washes in PBS to remove cell-unbound materials and additional cells culture for 48 h in GBM-free fresh medium (24 + 48 h). Representative optical images of cells exposed to FLG or d-GO showing materials sediments above cells are available in Supplementary materials (Figure S1).

### 2.3. Cell Viability

The effects of GBMs on cell viability were evaluated by the WST-8 reduction assay, based on the 2-(2-methoxy-4-nitrophenyl)-3-(4-nitrophenyl)-5-(2,4-disulfophenyl)-2H-tetrazolium probe, as previously described [12]. Briefly, cells exposed to GBMs were washed thrice before incubation for 4 h with WST-8-containing medium (10% *v*/*v*). As a positive control, cells were exposed to 5% sodium dodecyl sulphate (SDS). Absorbance was subsequently determined at 450 nm by an Automated Microplate Reader EL 311s (Bio-Tek Instruments; Winooski, VT, USA). Data are reported as % cell viability as compared to negative control (cells not exposed to GBMs).

### 2.4. Mitochondrial Damage

Mitochondrial damage was evaluated by means of mitochondrial depolarization using the JC-1 Mitochondrial Staining Kit (Sigma-Aldrich; Milan, Italy), as previously described [13]. Briefly, cells exposed to GBMs were washed thrice before incubation with 100 μL per well of 0.5 μM JC-1 probe for 20 min at 37 °C. As a positive control, 0.1 μg/mL valinomycin (Sigma-Aldrich; Milan, Italy) was used. Fluorescence was measured using a Fluorocount Microplate Fluorometer (Packard; Munich, Germany). Red fluorescence given by JC-1 aggregates indicates intact mitochondria and was detected with excitation and emission wavelengths of 530 and 590 nm, respectively. Green fluorescence given by JC-1 monomers indicates disrupted mitochondria and was measured with excitation and emission wavelengths of 485 nm and 570 nm, respectively. The ratio between red and green fluorescence intensity indicates mitochondrial depolarization. Data are reported as % of mitochondrial depolarization as compared to negative controls (cells not exposed to GBMs).

### 2.5. ROS Production

ROS production was evaluated using the 2′,7′-dichlorofluorescin diacetate (DCFDA) probe (Sigma-Aldrich; Milan, Italy), as previously described [13]. Briefly, HaCaT cells were stained with 100 μM DCFDA probe for 30 min at 37 °C in the dark and, after two washings, exposed to GBMs in complete medium without phenol red. As a positive control, cells were exposed to 0.3 mg/mL 2,2′-azobis(2-amidinopropane) dihydrochloride (AAPH; Sigma-Aldrich, Milan, Italy). Fluorescence was read using a Fluorocount Microplate Fluorometer (Packard; Munich, Germany) with excitation and emission wavelengths of 485 nm and 570 nm, respectively. Results are expressed as % of ROS production as compared to negative controls (cells not exposed to GBMs).

### 2.6. Autophagy Analysis (Western Blot)

To evaluate autophagic activity, HaCaT cells were exposed to 1–100 µg/mL GO or FLG for 24 h, and with or without 5 µM ammonium chloride for the last 3 h. Then, whole HaCaT cell proteins were extracted using Laemmli buffer, constituted by 125 mM Tris-HCl buffer pH 6.8, 2% (*w*/*v*) sodium dodecyl sulfate (SDS); 10% (*v*/*v*) glycerol and 5% (*v*/*v*) β-mercaptoethanol. Cell lysates were separated on a 4–20% gradient gel (Biorad), transferred onto a polyvinylidene difluoride (PVDF) membrane and blocked with PBS added with 0.1% (*v*/*v*) Tween 20 (PBS-T) and 5% (*w*/*w*) non-fat dry milk for 1 h. Membranes were then incubated for 50 min at 4 °C with 1 µg/mL primary antibodies in PBS-T containing 5% non-fat dry milk. The antibodies used were specific for the LC3 autophagic marker (Abcam; Cambridge, UK, #ab51520) and the β-actin loading control (Santa Cruz Biotechnology; Dallas, TX, USA, #47778). After washing with PBS-T, membranes were incubated for 30 min at room temperature with goat anti-rabbit IgG antibody (Southern Biotech; Birmingham, AL, USA, #1030-05) conjugated to horseradish peroxidase (HRP). The signal was detected using enhanced chemiluminescence detection reagents (Immobilon Western, Merck Millipore, Darmstadt, Germany, #WBKLS0500) and visualized on radiographic film in a Kodak processor (#M35-M X-OMAT, Rochester, NY, USA).

### 2.7. Transmission Electron Microscopy (TEM)

For TEM (Hitachi H600; Tokyo, Japan) imaging, HaCaT cells were cultured in 12-well plates at a density of 1 × 10^6^ cells per well and allowed to adhere before exposure to 100 µg/mL FLG for 24 h, along with control untreated cells. After incubation, the cells were washed twice with cacodylate buffer and fixed in 2.5% glutaraldehyde in cacodylate buffer at 4 °C overnight. Following overnight fixation, the cells were rinsed thrice with cacodylate buffer alone. Later, the cells were post-fixed with 0.5% osmium tetroxide for 30 min at room temperature and washed thrice with MilliQ^®^ water. Cells were then dehydrated through a series of ethanol baths: 1 × 25% ethanol for 10 min, 1 × 50% ethanol for 15 min, 1 × 70% ethanol for 15 min, 1 × 95% ethanol for 10 min and 3 × 100% ethanol for 15 min. Following dehydration, the cells were soaked in 1:1 ratio of 100% ethanol and Epon™ overnight at 4 °C. The next day, the cells were rinsed (2 × 4 h) with Epon™. After soaking, final inclusion of Epon™ into the cells was done by polymerizing Epon™ at 40 °C for 30 min then incubating at 60 °C for 48 h. Afterwards, the polymerized blocks were removed and sliced into ultrathin sections using a diamond knife attached to a ultramicrotome cutter (Leica; Wetzlar, Germany). The ultrathin sections were then collected on copper grids coated with Butvar^®^ B-98 and stained with 1% uranyl acetate for 30 min followed with lead citrate staining for 2 min. Grids were then examined by TEM (Hitachi H600; Tokyo, Japan). Control cells without FLG underwent the same procedure.

### 2.8. Statistical Analysis

Results of cytotoxicity assays are the mean ± standard error (SE) of at least 3 independent experiments performed in triplicate. Statistical analysis was performed by a one-way or two-way ANOVA followed by Bonferroni’s post-test (GraphPad Prism version 6.00; GraphPad Software; San Diego, CA, USA) and statistical significance was considered for *p* values < 0.05.

## 3. Results

### 3.1. Reversibility of GBM-induced Cytotoxicity

To investigate the reversibility of GBM-induced cytotoxicity toward HaCaT keratinocytes, cells were exposed to FLG or d-GO (1–100 µg/mL) for 24 or 72 h (continuative exposure), or for 24 h followed by 48 h culture without GBMs (recovery exposure; 24 + 48 h). Then, cell viability was evaluated by the WST-8 assay.

**Figure 1:**
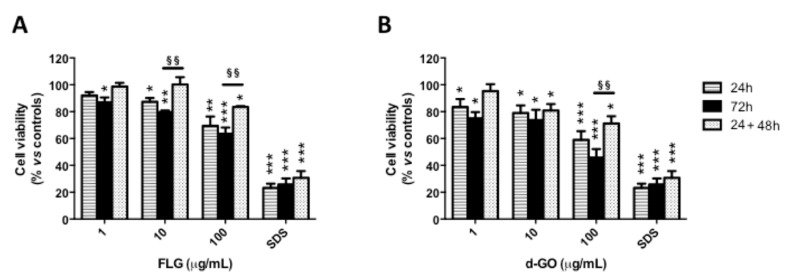
Cytotoxicity of graphene-based materials (GBMs) toward HaCaT cells. HaCaT cells were exposed to GBMs (1, 10 and 100 µg/mL few layer graphene (FLG; (**A**)) or graphene oxide (d-GO; (**B**))) for 24 or 72 h (continuous exposure), or for 24 h followed by 48 h recovery in GBM-free medium (recovery exposure; 24 + 48 h) before the evaluation of cell viability by the WST-8 assay. As a positive control, cells were exposed to 5% sodium dodecyl sulphate SDS. Results are the mean ± standard error (SE) of 3 independent experiments performed in triplicate. Statistical differences vs. negative controls: *, *p* < 0.05; **, *p* < 0.01; ***, *p* < 0.001 (One-way ANOVA and Bonferroni’s post-test). Statistical differences between continuative and recovery conditions: ^§§^, *p* < 0.01 (Two-way ANOVA and Bonferroni’s post-test).

As depicted in Figure 1, continuous cell exposure to FLG or d-GO for 24 or 72 h induced a significant concentration-dependent reduction of cell viability. As compared to negative controls, 24 h exposure to the highest FLG or d-GO concentration significantly reduced cell viability at 69% (*p* < 0.01) and 59% (*p* < 0.001), respectively. The cytotoxic effect of 24 h exposure to FLG or d-GO was slightly, but not significantly (*p* > 0.05), reverted culturing the cells for further 48 h in GBM-free conditions (recovery exposure; 24 + 48 h). In addition, cell viability recorded after 24 h exposure to FLG (10 and 100 µg/mL) or d-GO (100 µg/mL) followed by 48 h recovery in GBM-free medium (recovery exposure; 24 + 48 h) was significantly higher than that recorded after continuous exposure to GBMs for 72 h. In particular, as compared to negative controls, viable cells after exposure to 10 and 100 µg/mL FLG under the recovery condition (24 + 48 h) were 99% (*p* < 0.01) and 84% (*p* < 0.01), respectively, significantly higher than those recorded after 72 h continuous exposure to the same FLG concentrations (79 and 63%, respectively; *p* < 0.01). As compared to negative controls, the viability of cells exposed to 100 µg/mL d-GO under the recovery condition (24 + 48 h; 72%) was significantly higher than that recorded after 72 h continuous exposure (46%, *p* < 0.01).

These data suggest that both FLG and d-GO induce a cytotoxic effect toward HaCaT keratinocytes already after 24 h exposure, which can be partially reverted upon the removal of GBMs, in particular FLG.

### 3.2. Reversibility of GBM-Induced Mitochondrial Damage

To investigate the reversibility of GBM-induced mitochondrial damage, HaCaT cells were exposed to FLG or d-GO (1–100 µg/mL) for 24 or 72 h (continuous exposure), or for 24 h followed by 48 h culture in GBM-free medium (recovery exposure; 24 + 48 h). Then, mitochondrial damage was evaluated by means of mitochondrial depolarization using the potentiometric JC-1 probe.

**Figure 2:**
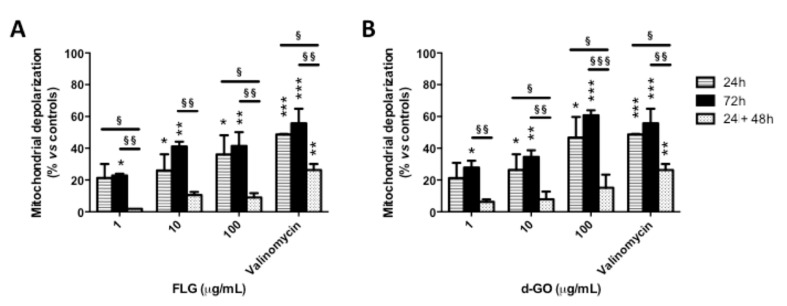
Effect of GBMs on mitochondrial depolarization in HaCaT cells. HaCaT cells were exposed to FLG (**A**) or d-GO (**B**) (1, 10 and 100 µg/mL) for 24 or 72 h (continuous exposure), or for 24 h followed by 48 h recovery in GBM-free medium (recovery exposure; 24 + 48 h) before the evaluation of mitochondrial depolarization using the JC-1 potentiometric probe. As a positive control, cells were exposed to 0.1 μg/mL valinomycin. Results are the mean ± SE of 3 independent experiments performed in triplicate. Statistical differences vs. negative controls: *, *p* < 0.05; **, *p* < 0.01; ***, *p* < 0.001 (One-way ANOVA and Bonferroni’s post-test). Statistical differences between continuative and recovery conditions: ^§^, *p* < 0.05; ^§§^, *p* < 0.01; ^§§§^, *p* < 0.001 (Two-way ANOVA and Bonferroni’s post-test).

As shown in Figure 2, continuous exposure to FLG or d-GO for 24 or 72 h increased mitochondrial depolarization in a concentration-dependent manner. As compared to 24 h exposure to GBMs, cells exposed to FLG (1 and 100 µg/mL) or d-GO (10 and 100 µg/mL) for 24 h followed by 48 h recovery in GBM-free medium (recovery exposure; 24 + 48 h) showed a significant reversion of the mitochondrial depolarization. In particular, as compared to negative controls, cell exposure to 1 and 100 µg/mL FLG for 24 h increased mitochondrial depolarization at 21 and 36%, respectively, which was significantly reduced to 2% (*p* < 0.05) and 9% (*p* < 0.05), respectively, if cells were cultured for a further 48 h under the recovery condition (24 + 48 h). Similarly, 24 h exposure to 10 and 100 µg/mL d-GO increased mitochondrial depolarization at 26 and 47%, respectively, which was significantly reduced to 8% (*p* < 0.05) and 15% (*p* < 0.05), respectively, under the recovery condition (24 + 48 h).

Mitochondrial depolarization induced by 24 h exposure to GBMs followed by 48 h recovery in GBM-free medium (recovery exposure; 24 + 48 h) was significantly lower than that induced by continuous exposure to the materials for 72 h. In particular, as compared to negative controls, 72 h exposure to 1, 10 and 100 µg/mL FLG increased mitochondrial depolarization at 22, 41 and 41%, respectively, significantly higher than that induced under the recovery condition (24 + 48 h), corresponding to 2% (*p* < 0.01), 10% (*p* < 0.01) and 9% (*p* < 0.01), respectively. Cells exposed to 1, 10 and 100 µg/mL d-GO for 72 h showed increased mitochondrial depolarization at 28, 35 and 60%, respectively, significantly higher than that recorded under the recovery condition (24 + 48 h): 6% (*p* < 0.01), 8% (*p* < 0.01) and 15% (*p* < 0.001), respectively.

These results suggest that keratinocytes are able to revert the effect of GBMs on mitochondrial depolarization upon the removal of these materials, in particular FLG.

### 3.3. Reversibility of GBM-induced ROS Production

To investigate the reversibility of GBM-induced ROS production, HaCaT cells were exposed to FLG or d-GO (1–100 µg/mL) for 24 or 72 h (continuative exposure), or for 24 h followed by 48 h recovery in GBM-free medium (recovery exposure; 24 + 48 h). Then, ROS production was evaluated by the DCFDA fluorescence dye.

**Figure 3:**
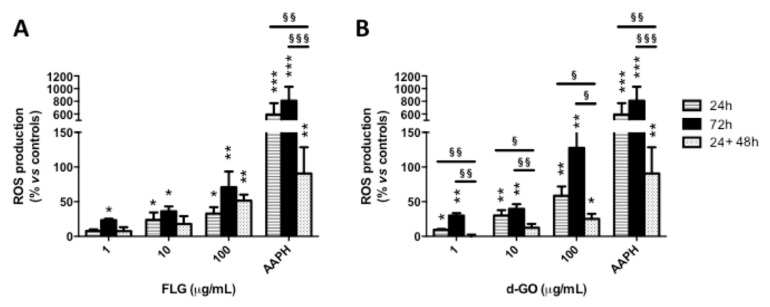
Reactive oxygen species (ROS) production by HaCaT cells exposed to GBMs. HaCaT cells were exposed to FLG (**A**) or d-GO (**B**) (1, 10 and 100 µg/mL) for 24 or 72 h (continuous exposure), or for 24 h followed by 48 h recovery in GBM-free medium (recovery exposure; 24 + 48 h) before the evaluation of ROS production using the 2′,7′-dichlorofluorescin diacetate (DCFDA) fluorescence probe. As a positive control, cells were exposed to 0.3 mg/mL 2,2′-azobis(2-amidinopropane) dihydrochloride (AAPH). Results are the mean ± SE of 3 independent experiments performed in triplicate. Statistical differences vs. negative controls: *, *p* < 0.05; **, *p* < 0.01; ***, *p* < 0.001 (One-way ANOVA and Bonferroni’s post-test). Statistical differences between continuative and recovery conditions: ^§^, *p* < 0.05; ^§§^, *p* < 0.01; ^§§§^, *p* < 0.001 (Two-way ANOVA and Bonferroni’s post-test).

As depicted in Figure 3, cell exposure to each material for 24 or 72 h increased ROS production in a concentration-dependent manner, with the highest effect after 72 h. As compared to GBM exposure for 24 h, culturing the cells for a further 48 h in GBM-free medium (recovery exposure; 24 + 48 h) showed that only d-GO-induced ROS production was significantly reverted, at all the concentrations tested. In particular, as compared to negative controls, 24 h exposure to 1, 10 and 100 µg/mL d-GO increased ROS production at 10, 30 and 59%, respectively, which was reduced under the recovery condition (24 + 48 h) to 1% (*p* < 0.01), 12% (*p* < 0.05) and 25% (*p* < 0.05), respectively.

In addition, d-GO-induced ROS production induced by each concentration after the recovery condition (24 + 48 h) was significantly lower than that induced by 72 h continuous exposure to this material, whereas no significant difference was recorded for FLG. In particular, as compared to negative controls, 72 h exposure to 1, 10 and 100 µg/mL d-GO increased ROS production at 30, 40 and 128%, respectively, levels significantly higher than those detected under the recovery condition (24 + 48 h): 1% (*p* < 0.01), 12% (*p* < 0.01) and 25% (*p* < 0.05), respectively.

These results suggest that ROS production induced by d-GO is significantly reduced in HaCaT cells upon its removal from culture medium, whereas no reversible effect was observed for FLG.

### 3.4. Effects on Autophagy

Autophagy is an important intracellular recycling pathway that might be affected by the presence of foreign materials, including GBMs. Autophagic proteins, such as LC3-II, serve as a readout of autophagy, although an increase can signify either an accumulation due to blockage or an increased flux through increased cycling of autophagy. Autophagic flux can then be calculated by the difference between LC3-II/actin immunoblotted proteins in a sample treated with the lysosome inhibitor ammonium chloride (ACH) and the corresponding untreated sample.

**Figure 4:**
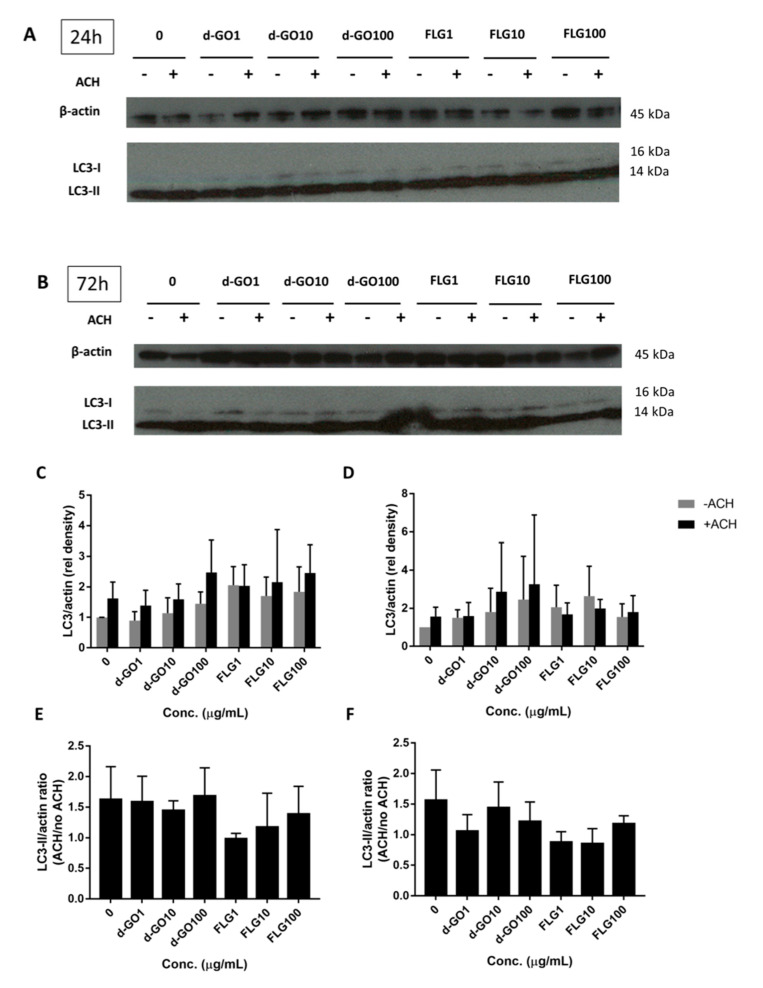
Autophagy effects in HaCaT cells exposed to GBMs. HaCaT cells were exposed to GBMs (1, 10 and 100 µg/mL FLG or d-GO) for 24 or 72 h (continuous exposure) before the evaluation of autophagic flux. (**A**,**C**,**E**) 24 h exposure and (**B**,**D**,**F**) 72 h exposure. Autophagic flux (**E**,**F**) is the ratio of LC3-II/actin (**C**,**D**) treated with ammonium chloride (ACH) over LC3-II/actin not treated with ACH. Figure 4E (relative density graph of Figure 4A) was derived from Figure 4C, while Figure 4F (relative density graph of Figure 4B) was derived from Figure 4D. LC3-II bands are the thicker, lower bands. All results were non-significant.

To better understand the impact of GBMs on autophagy, HaCaT cells were continuously exposed for either 24 or 72 h with 1, 10 and 100 µg/mL FLG or d-GO, before harvesting and blotting with LC3 antibodies. (Figure 4A,B) The ratio of LC3-II/actin (Figure 4C,D) and autophagic flux (Figure 4E,F) were then calculated.

All results were non-significant, with no change in the autophagic flux for d-GO and a non-significant trend to decrease the autophagic flux for FLG (Figure 4E,F).

### 3.5. GBM Cell Internalization

**Figure 5:**
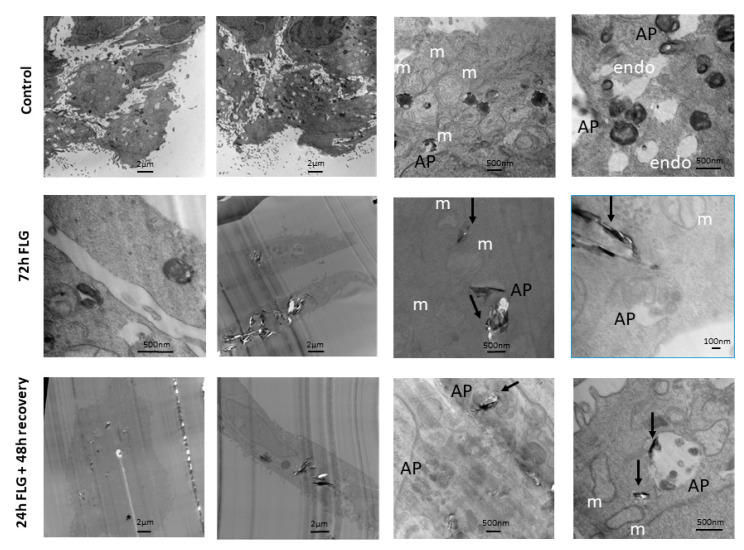
Transmission electron microscopy (TEM) of HaCaT cells exposed to FLG. HaCaT cells were exposed to FLG (100 µg/mL) for 24 or 72 h (continuous exposure) or for 24 h followed by 48 h recovery in GBM-free medium (recovery exposure; 24 + 48 h) before fixation and preparation for TEM. The panels on the left half show almost entire cells at lower magnification, while the panels on the right half show cells at higher magnification. (m, mitochondria, AP, autophagosome, endosome).

Given that the major reversibility of cytotoxicity was observed in case of FLG, cell internalization analysis was performed considering this material. In control untreated cells (Figure 5, top raw), phagosomes containing splotchy dark material were found in some cells. Typical intracellular keratinocyte landmarks such as branched keratin fibers and abundant mitochondria were also observed. Autophagosomes containing multi-membraned organelles were seen. These resembled cells with dark peripheral edges when viewed at lower magnification.

For this experiment, we focused our attention on FLG. In the FLG 72 h-treated cells (Figure 5, middle raw), FLG led to tearing of cell slices, leading to a bias in cell selection, as cells containing plenty of FLG tended to be ripped apart easily, and hence not available for observation. No abnormal mitochondria or unusual cell organelles were observed. FLG of varying sizes were taken up into phagosomes deep in the cell, singly and in multiples. Dense dark non-FLG materials as well as FLG, were found engulfed in autophagosomes.

Lastly, in the FLG 24 + 48h recovery cells (Figure 5, bottom raw), FLG of varying sizes were taken up into phagosomes deep in the cell, singly and in multiples. No abnormal mitochondria or unusual cell organelles were observed. Autophagosomes containing multi-membraned organelles were seen. These sometimes contained FLG as well. Empty phagososomes, which did not contain dark material, were also visible.

## 4. Discussion

In the frame of GBM hazard characterization, particular attention should be given to the effects induced by cutaneous exposure. In fact, skin contact is one of the most relevant exposure routes to these materials, considering both an occupational scenario and specific ones related to skin applications of GBMs [8,9]. The latter consists of GBM-enriched devices used as biosensors, wearable soft bioelectronics and novel touchscreens, but can be represented also by GBM-enriched novel textiles, seat coatings and even new-generation condoms, among others [19,20,21,22]. In this regard, we already characterized the in vitro toxic effects induced by short (24 h) and long (72 h) exposures to GBMs on skin HaCaT keratinocytes, a spontaneously immortalized non-tumor cell line that, resembling the functional properties of undifferentiated keratinocytes, is widely used as a screening tool to assess the mechanism of cutaneous toxicity [23]. In HaCaT cells, GBMs significantly reduced cell viability only after long exposure times (i.e., 72 h) at high concentrations (>10 μg/mL) [12,13], suggesting that keratinocyte exposure to GBMs can be detrimental only after high exposure conditions. In addition, we have previously demonstrated that GBM effects are dependent upon a sustained interaction with the plasma membrane, which appears to be quite stable [12]. However, no information is available on a possible reversibility of this damage, which could have a significant positive impact on the safety of devices involving skin contact to high concentrations of GBMs, even for a limited period of time. To the best of our knowledge, the assessment of the reversibility of GBM toxicity, if any, is not a frequent approach in this field.

Hence, the reversibility of in vitro GBM effects towards HaCaT skin keratinocytes was investigated exposing, the cells to the materials for a short exposure time (24 h) and evaluating the recovery of the damage after their removal. To this aim, we used a research grade FLG and a d-GO, as representative of GBMs. Both materials were endotoxin-free, avoiding result alteration due to false positive data given by LPS contamination, a common feature of GBMs [24,25]. Intriguingly, the same endotoxin-free materials were shown to be sensed by keratinocytes after both continuative and recovery exposures: keratinocytes exposed to low concentrations (0.01–1.0 μg/mL) of FLG or d-GO for a short exposure time (4 h) followed by longer culture in GBM-free medium (20 up to 68 h) significantly released pro-inflammatory cytokines, and this release appeared to be significantly higher compared to the corresponding continuous exposure to the materials for 24 or 72 h [15]. This result suggests that, at least considering pro-inflammatory cytokine release, an initial GBM detection is sufficient to activate HaCaT keratinocytes, and that GBM removal is not able to recover cells from the pro-inflammatory stimulus. However, cytokine release is a complex phenomenon that, even though activated, does not necessarily lead to a complete adverse reaction, such as inflammation. Indeed, in the same study we demonstrated that, under recovery conditions, despite FLG and GO inducing a pro-inflammatory cytokine release by keratinocytes, this event was not sufficient to activate monocytes as a subsequent step in the skin inflammation process [15].

On the basis of these considerations, herein, we investigated if the cytotoxic effects of high FLG and d-GO concentrations in HaCaT cells can be reverted by the material’s removal, as an in vitro approach to investigate the reversibility of GBM damage. To this aim, we compared the effects of GBMs under a recovery condition (24 h cells exposure to GBMs followed by 48 h culture in GBM-free media) to those induced by continuous cell exposure to the materials for 24 or 72 h. FLG and d-GO were tested at concentrations previously shown to induce a significant cytotoxic effect (1–100 µg/mL) in HaCaT skin keratinocytes [12], and a panel of cellular parameters was evaluated. These parameters were chosen on the basis of the cellular functionalities altered by GBMs in keratinocytes, such as cell viability, mitochondrial depolarization and ROS intracellular levels [13]. Considering the first two parameters, in general our results show that both FLG and d-GO induced a cytotoxic effect toward HaCaT keratinocytes already after 24 h exposure, and that keratinocytes were able to partially revert this effect upon GBM removal, in particular FLG. This effect appears to be more evident considering the mitochondrial damage: mitochondrial depolarization in HaCaT cells exposed to each material was reverted upon their removal, in particular of FLG. FLG effect under the recovery condition (24 h exposure followed by 48 h cells culture in FLG-free medium) appeared to be significantly lower, not only in comparison to the corresponding continuous exposure (72 h), but, most importantly, in comparison to 24 h of exposure. This observation suggests a partial reversibility of GBM effects at the mitochondrial level, even if it appears to be incomplete. As expected, removal of the positive control valinomycin implied a significant recovery of mitochondrial depolarization. On the contrary, removal of SDS, the positive control for the WST-8 assay, did not imply recovery of the cytotoxic effect; this was not surprising given the quick necrotic-like damage induced by this substance.

Considering ROS production, a different behavior was found between FLG and d-GO. In particular, only ROS production by HaCaT cells exposed to d-GO was significantly reduced upon the material removal from culture medium, whereas no significant effect was observed in case of FLG removal. This observation suggests that only d-GO induced oxidative stress is a partially reversible cytotoxicity parameter. This conclusion could rely on the different oxidative capabilities of the two materials, d-GO being more potent in inducing ROS production, probably due to the higher density of O_2_-bearing groups in comparison to FLG [13,26]. Additionally, in this case, removal of the positive control AAPH implies a recovery from ROS production.

In general, the results of this study demonstrate that GBMs effects on skin keratinocytes are only partially reversible. This conclusion is particularly evident for FLG, probably because of its slightly lower cytotoxicity in comparison to d-GO on these cells [12,13]. The incomplete reversibility of GBM effects observed in our study could be also ascribed to the capability of keratinocytes to internalize the materials, which could exert their effects even after their physical removal from the cell medium. Indeed, as shown by TEM analysis, intracellular GBM depots were detected. This observation leads us to speculate that, even if the majority of the unbound material is washed out from cell media in the recovery condition, the rate of bound and/or internalized GBMs is sufficient to exert a cytotoxic effect.

Under recovery conditions, such as those employed in our study, a key phenomenon able to recover cells from damages is represented by autophagy. Indeed, autophagy plays an important role in mediating cell survival and maintenance, through the degradation and recycling of cytoplasmic organelles, proteins, and macromolecules. The role of autophagy in the mechanism of nanomaterial toxicity has been well-reviewed by many groups [27,28]. In this context, we embarked on the possibility of this important intracellular pathway playing a role in toxicity and its reversibility with our graphene materials. For instance, GO nanoribbons were found to induce autophagic vacuoles in neuroblastoma cell lines [29], although we did not observe any autophagy-related changes involving d-GO, which has been previously reported in macrophages to induce autophagy [30]. The same authors alluded to the importance of physical characteristics of the material on the autophagic effect, as they found that single-walled carbon nanotubes had a lower impact on autophagy than GO. This could similarly be the case in our study, given that many other nanomaterial factors such as the method of preparation, size, presence of functional groups and material stability, could play a role. Western blot upregulation of the autophagosome marker LC3-II was previously reported with non-toxic concentrations of titanium dioxide nanoparticles in HaCaT cells [31], which we have also observed. However, that study did not use concomitant bafilomycin or ammonium chloride to inhibit LC3-II breakdown, as a method to gauge autophagic flux, which is related to turnover. As such, although we report no significant change in overall autophagy, we provide a more rounded analysis with regards to autophagic flux. Interestingly, the same study concluded that titanium dioxide nanoparticles shifted from autophagy induction to blockage, which is in line with our data on decreased autophagic flux with FLG. Another group working on carbon nanofibers in lung epithelial cells reported impaired lysosomal function and cytoskeleton disruption mediated by an autophagic flux blockade as a major cause of accumulation rather than autophagy induction, which activates apoptosis, relating it to increased ROS levels [32]. This could be a new direction to explore, given that our study focused more on autophagy rather than lysosomal pathways.

Like what other researchers have reported with FLG in cellular TEM [33], there is a tendency for the presence of FLG to cause tearing of cell slices, inadvertently causing white space in the captured image and a decrease in image contrast, thereby losing visibility of many less prominent cell features such as keratin fibers. This has hindered more in-depth observations, like in other HaCaT studies showing autophagy induction, but which have featured non-graphene materials [34,35]. Compared to the untreated control, material from both 72 h FLG and 24 h FLG + 48 h recovery could be observed to be taken up by phagosomes unevenly without causing major morphological abnormalities. This means that the mere exposure of FLG was sufficient to lead to irreversible cell uptake, although the lack of aberrations showed that this was not completely detrimental, in line with the partial reversibility of cytotoxicity. Having observed a variety of organelles with TEM, we defined primary lysosomes as uniformly dark and round, and secondary lysosomes (heterolysosomes) as fused with endosomes and generally containing other cellular organelles, but not FLG. Endosomes were defined as present at the cell periphery and empty, while autophagosomes observed in general had a dark peripheral cluster of membranes. The observed uptake by phagosomes and not lysosomes and autophagosomes was expected, although it showed that autophagy did not play a major role. Taking into consideration that different materials have different effects on different cell types, this could be also due to the relatively short duration (72 h) of treatment and relatively low concentrations used that may be material-specific to see an effect.

In conclusion, the results of this study demonstrate a partial reversibility of GBM-induced cytotoxicity in skin HaCaT keratinocytes. In particular, recovery experiments suggest that the cytotoxicity induced by 24 h exposure to GBMs is only partially reduced by the material’s removal followed by culturing of cells for additional 48 h. This partial recovery could be ascribed to the material internalization by cells, which could allow them to continue their cytotoxic effect, even if with a slightly lower potency. In addition, this partial reversibility seems to be not mediated by autophagy activation, as a key phenomenon in mediating cellular damage recovery. Altogether, these results represent a step forward to characterize the hazard potential of GBMs at the skin level. In particular, the ability of GBMs to be internalized inside keratinocytes together with the partial reversibility of the cellular damage acquires a significant importance in assessing the risk associated with skin exposure to GBM-enriched devices, especially in the case of possible GBMs release.

## Supplementary Materials

The following are available online at https://www.mdpi.com/2079-4991/10/8/1602/s1, Figure S1: Representative optical image of HaCaT cells exposed to FLG or d-GO (1–100 μg/mL) for 24 h.
