# Supplementary material for: Partial Reversibility of the Cytotoxic Effect Induced by Graphene-Based Materials in Skin Keratinocytes
Source: Nanomaterials (Basel). 2020 Aug 15;10(8):1602. doi: 10.3390/nano10081602 (PMC7466707; doi:10.3390/nano10081602)
Supplement: Supplementary file 1 [file nanomaterials-10-01602-s001.pdf]

nanomaterials

Article

Partial Reversibility of the Cytotoxic Effect Induced by Graphene-based Materials in Skin Keratinocytes

Marco Pelin [1,*], Hazel Lin [2], Arianna Gazzi [3,4], Silvio Sosa [1], Cristina Ponti [1], Amaya Ortega [5], Amaia Zurutuza [5], Ester Vázquez [6,7], Maurizio Prato [3,8,9], Aurelia Tubaro [1] and Alberto Bianco [2,*]

1 Department of Life Sciences, University of Trieste, 34127 Trieste, Italy; mpelin@units.it; ssosa@units.it (S.S.); cponti@units.it (C.P.); tubaro@units.it (A.T.)
2 CNRS, Immunology, Immunopathology and Therapeutic Chemistry, UPR 3572, University of Strasbourg, ISIS, 67000 Strasbourg, France; rhlin@ibmc-cnrs.unistra.fr (H.L.)
3 Department of Chemical and Pharmaceutical Sciences, University of Trieste, 34127 Trieste, Italy; arianna.gazzi@phd.units.it (A.G.); prato@units.it (M.P.)
4 Fondazione Istituto di Ricerca Pediatrica, Città della Speranza, 35100 Padua, Italy
5 Graphenea, 20009 Donostia-San Sebastián, Spain; a.ortega@graphenea.com (A.O.); a.zurutuza@graphenea.com (A.Z.)
6 Facultad de Ciencias y Tecnologías Químicas, Universidad de Castilla-La Mancha (UCLM), 13071 Ciudad Real, Spain; Ester.Vazquez@uclm.es
7 Instituto Regional de Investigacion Científica Aplicada (IRICA), Universidad de Castilla-La Mancha, 13071 Ciudad Real, Spain
8 Center for Cooperative Research in Biomaterials (CIC biomaGUNE), Basque Research and Technology Alliance (BRTA), Paseo de Miramon 182, 20014 Donostia San Sebastián, Spain
9 Basque Foundation for Science (IKERBASQUE), 48013 Bilbao, Spain
* Correspondence: mpelin@units.it; Tel.: +39-040-5588620 (M.P.); A.Bianco@ibmc-cnrs.unistra.fr; Tel.: +33-388-417026 (A.B.)

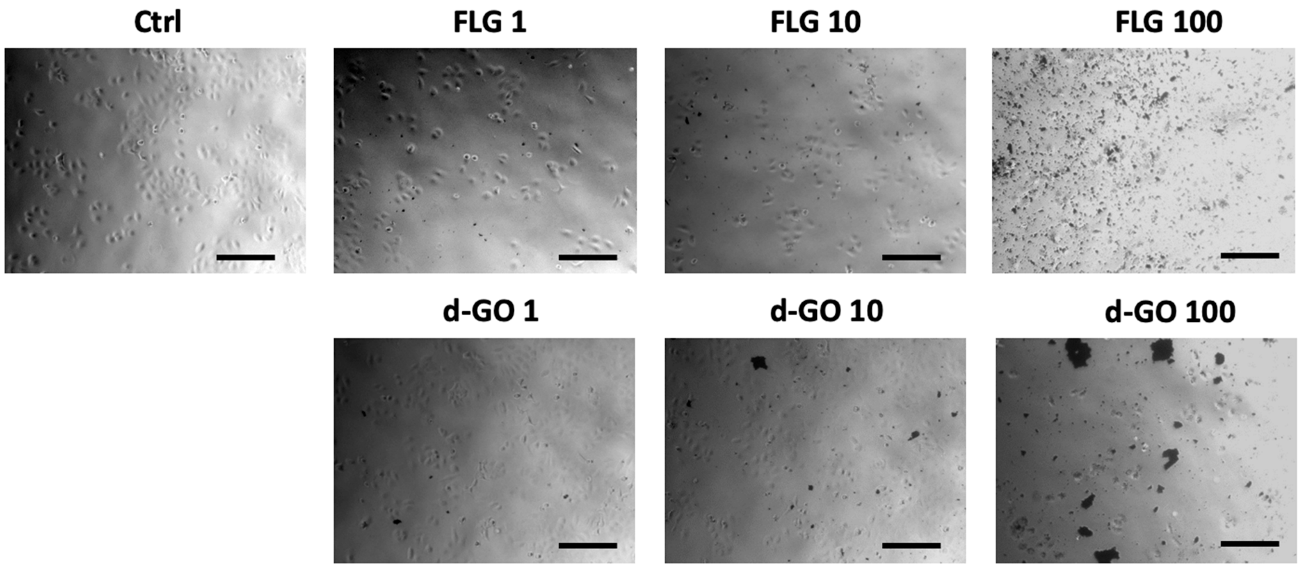

Figure S1. Representative optical image of HaCaT cells exposed to FLG or d-GO (1–100 µg/mL) for 24 h. Black dots represent aggregated materials deposited above cells. Magnification: 40×; scale bar: 20 µm.
